# Knowledge and perceptions of diabetes in a semi-urban Omani population

**DOI:** 10.1186/1471-2458-8-249

**Published:** 2008-07-22

**Authors:** Mohammed A Al Shafaee, Sulaiman Al-Shukaili, Syed Gauher A Rizvi, Yahya Al Farsi, Mushtaq A Khan, Shyam S Ganguly, Mustafa Afifi, Samir Al Adawi

**Affiliations:** 1 Department of Family Medicine and Public Health, College of Medicine and Health Sciences, Sultan Qaboos University, Oman; 2 Department of Non-Communicable Diseases, Ministry of Health (HQ), Oman; 3 Department of Behavioural Medicine, College of Medicine and Health Sciences, Sultan Qaboos University, Oman

## Abstract

**Background:**

Diabetes mellitus is a major public health problem in the Sultanate of Oman. This study aimed to evaluate the knowledge and perception of diabetes in a sample of the Omani general population, and the associations between the elements of knowledge and perception, and socio-demographic factors.

**Methods:**

The study was carried out in two semi-urban localities. A total of 563 adult residents were interviewed, using a questionnaire specifically designed for the present study. In addition to demographic information, the questionnaire contained questions on knowledge related to diabetes definition, symptoms, risk factors, complications and preventative measures, as well as risk perception for diabetes.

**Results:**

Knowledge of diabetes was suboptimal. The percentages of correct responses to questions on diabetes definition, classical symptoms, and complications were 46.5%, 57.0%, and 55.1%, respectively. Only 29.5%, 20.8% and 16.9% identified obesity, physical inactivity and a positive family history, respectively, as risk factors for diabetes. A higher level of education, a higher household income, and the presence of a family history of diabetes were found to be positively associated with more knowledge.

**Conclusion:**

This study demonstrated that there is   lack of awareness of major risk factors for diabetes mellitus. Level of education is the most significant predictor of knowledge regarding risk factors, complications and the prevention of diabetes. Given that the prevalence of diabetes has increased drastically in Oman over the last decade, health promotion seems essential, along with other means to prevent and control this emerging health problem.

## Background

Diabetes mellitus (DM) continues to be a major threat to global public health [1,2]. More than 170 million people worldwide have diabetes, and this figure is projected to more than double by the year 2030, if current trends continue [3]. The global increase in diabetes is triggered by, and associated with many factors, including the ageing population, and the unhealthy diets and sedentary lifestyles that heighten one's propensity towards obesity. In the industrialized countries of the West, diabetes is common among the elderly, in contrast with developing countries where diabetes most frequently affects those between the ages of 35 and 64 [4]. In some countries, DM also frequently occurs in youths [5].

The lack of an infrastructure for diabetic screening and high-risk group identification, in addition to inadequate public awareness and knowledge of diabetes symptoms may explain the failure of early diagnosis and, as a consequence, the burdens and loss of economic output associated with diabetes. There is growing evidence that preventing and/or delaying the onset of diabetes is a viable option [6,7]. Increased physical activity, modest weight reduction, and pharmacological interventions can decrease the incidence of diabetes complications significantly, even among high-risk groups. Simple lifestyle modifications, such as a healthy diet that includes reducing sugar intake, are considered to be essential for the prevention and control of incident diabetes mellitus [8-10]. Thus, increasing public awareness regarding modifiable diabetes risk factors and healthier lifestyles, and developing strategies to identify and manage at-risk populations, are among of the various possible mechanisms being used to stem the present epidemic of diabetes in many parts of the world.

The prevalence of diabetes in the Sultanate of Oman in 2000, as revealed by the *2000 National Health Survey*, was 11.6%, compared with 8.3% in 1991, representing an increase of 40% over a single decade [11]. Additional increases in diabetes prevalence are likely, in light of projected changes in overall population growth, continuous adverse changes in dietary habits, and increasing numbers of people who are overweight, obese, or less physically active. Higher prevalence rates of diabetes have been identified in more urbanized areas of the country (18%) relative to more rural areas (11%) [12].

It is widely accepted that many problems, previously thought of as primarily medical and, hence, demanding conventional medical intervention, are in fact more appropriately disentangled by changing individual and social attitudes and behaviors [13]. Many health promotion strategies have had only modest success, because prevailing knowledge and perceptions often seem to override biomedical assumptions and considerations [14]. Various recent studies conducted in many parts of the world suggest that there is a lack of public awareness and knowledge of various factors related to diabetes [15-17]. This study aims to evaluate the level of knowledge and overall perceptions of diabetes within the general population of Oman. To the best of our knowledge, no such study has been conducted previously in the Sultanate of Oman.

## Methods

### Study population

This study was carried out in two semi-urban villages. As part of the *Village Health Care Course*, provided by the College of Medicine and Health Sciences at Sultan Qaboos University, two villages – Al Rumais and Al-Shuaiba, located 60 km and 80 km north of the University, respectively – were selected to carry out this field study. These two semi-urban villages are adjacent to the metropolitan city of Muscat, which also is the capital city of Oman. The demographics of these two villages are at least somewhat representative of the ethnicity and different strata of Omani society, by virtue of them being satellite towns of the capital, which has recently attracted migration from different parts of the country [12].

After receiving administrative approval from local authorities, as well as ethics approval from the *College of Medicine & Health Sciences*, all households in the two villages were visited, whereupon information regarding the nature and general purpose of the study was provided. Those households in which informed consent to participate was granted were included in this study. Within each household, all persons 20 years of age or older were included in the sampling frame, which ultimately was comprised of 628 eligible persons. If any eligible household member was not available to complete the interview, arrangements were made for a follow-up interview. A maximum of three attempts were made to contact each eligible person during the survey period, which spanned from the 6^th ^to 14^th ^of January, 2007. A total of 563 eligible adults were interviewed, representing a response rate of 89.6%. When there were multiple respondents from the same household, they were interviewed separately to avoid peer or family influence. Individuals who reported a history of diabetes mellitus among first-degree relatives were considered to have a positive family history.

### Measurement

A review of the literature concerning different aspects of public knowledge and perceptions towards diabetes identified potential items to include in the survey questionnaire used in this study [18,19]. The final survey instrument contained 24 items, subdivided into 5 sections. The first two sections included questions on participant demographics and medical history. The third section was intended solely for diabetic participants and covered their diabetic history and glycemic control status. Knowledge regarding diabetes definition, risk factors, signs and symptoms, and complications was examined in the fourth section. The last section concentrated on the perceived risk of developing diabetes, as well as the participant's perception regarding diabetes prevalence, prevention and community awareness.

The questionnaire used Likert-type response scales. In order to collect additional data otherwise unobtainable with a typical Likert scale, open-ended questions were included, as well. For example, the closed-ended question – "Do you use your treatment regularly?" – was followed by the open-ended question – "If no, what are the reasons?"

The questionnaire was pre-tested and piloted within a convenience sample of students and staff at the College of Medicine and Health Sciences at Sultan Qaboos University. Its psychometric properties were found to be adequate. For consistency, and to accommodate illiterate subjects and those with sensory and motor deficits, the questionnaires were read out loud to the subjects, rather than being self-administered. The interviews were conducted by 15 trained researchers, predominantly 3rd and 4th year medical students from the College of Medicine and Health Sciences at Sultan Qaboos University. During our preparation for this study, the interviewers were trained to read out the items of the questionnaire clearly and consistently, and to code the responses with precision and reliability; we observed substantial inter-coding agreement for the scale items (r = 0.86, p < 0.001).

### Statistical analysis

The association between study variables assessing (1) components of risk factor-related knowledge (e.g., excessive sugar intake, obesity, reduced physical activity, the presence of a family history); and (2) preventative measures (e.g., healthy dietary practises, increasing physical activity and avoiding obesity); and (3) certain socio-demographic characteristics (e.g., participant gender, age, educational status, monthly income and family history of diabetes) were determined by estimating the difference in proportions, using Pearson's chi-square analysis. The relationship between the various components of knowledge (i.e., disease definition, symptoms, risk factors, complications and preventive measures) and socio-demographic factors were evaluated separately using adjusted odds ratios with 95% confidence intervals. To accomplish this, a binary logistic regression model utilizing the step-wise backward conditional method was created and tested for each component. For this, the knowledge of the individual was considered as the dependent variable and was coded "1" if the respondent was found to be aware and "0" otherwise. Odds ratios were assessed for significance using Wald analysis. All significance tests were two-tailed, and a probability value of less than 0.05 was considered statistically significant. All data variables were processed, and analysis was performed using SPSS version 10 for Windows.

## Results

A total of 563 individuals were surveyed. Demographic and clinical characteristics of the subjects and gender differences are shown in Table 1. It can be observed that 86.1% of the subjects were below the age of 50 years. About forty-six percent had studied up to high school or college levels. A positive family history of diabetes and personal diabetes were reported by 53.8% and 6.6% of the subjects, respectively. No statistically significant gender differences in demographic characteristics were identified (p > 0.05) in terms of self-reported diabetes, hypertension, or family history of diabetes.

**Table 1:** Demographic characteristics of the sample and gender differences

	**Males**		**Females**		**Total**		**p-value**^+^
	(n = 237)	42.1%	(n = 326)	57.9%	(n = 563)	100%	
Age group							0.023
20–30 yrs.	128	54.0	170	52.2	298	52.9	
31–50 yrs.	67	28.3	120	36.8	187	33.2	
> 50 yrs.	42	17.7	36	11.0	78	13.9	

Educational status							0.030
Illiterate	34	14.3	78	23.9	112	19.9	
Less than high school	84	35.4	105	32.2	189	33.6	
High school	72	30.4	95	29.2	167	29.6	
Some college or more	47	19.8	48	14.7	95	16.9	

Monthly household income							0.005
Less than 300 OMR*.	72	31.3	139	44.7	211	39.0	
300 – 1000 OMR.	126	54.8	143	46.0	269	49.7	
More than 1000 OMR.	32	13.9	29	09.3	61	11.3	

Diabetes status- self reported							0.567
Diabetic	14	05.9	23	07.1	37	06.6	
Non-diabetic	223	94.1	303	92.9	526	93.4	

Hypertension status- self reported							0.135
Hypertensive	21	08.9	42	12.9	63	11.2	
Non-hypertensive	216	91.1	284	87.1	500	88.8	

Family history of diabetes							0.196
Positive history	120	50.6	183	56.1	303	53.8	
No OR not sure	117	49.4	143	43.9	260	46.2	

* OMR = Omani Rial which is 2.6 to US Dollar							

+p-values for comparisons between the gender difference

Three hundred and twenty subjects (56.8%) reported that they were aware of the meaning of the condition called *diabetes*. However, when they were asked to define it, only 262 subjects (46.5%) were able to give at least a rudimentary definition (data not shown in the table). Most frequently, diabetes is defined as 'a disease in which there are elevated levels of sugar in the blood'.

Three hundred and twenty one of the subjects (57.0%) knew at least one of the classical symptoms of diabetes, like polyuria, polydipsia or unexplained weight loss. Polyuria was the most commonly identified symptom reported by 252 subjects (44.8%) followed by unexplained weight loss 139 (24.7%), polydipsia 113 (20.1%), lethargy 64 (11.4%) and giddiness 62 (11.4%). A significant gender differential was noticed with respect to identifying unexplained weight loss, giddiness and malaise as symptoms of diabetes (p < 0.05) (data not shown in the table).

Rates of awareness regarding important diabetes risk factors – like excessive sugar intake, obesity, physical inactivity and the presence of a family history – are shown in Table 2. About 59.9% perceived high consumption of dietary sugar as an important risk factor for developing diabetes. Only 29.5%, 20.8% and 16.9% perceived obesity, physical inactivity and positive family history, respectively, as risk factors for diabetes. Awareness of physical inactivity as a risk factor was more common among male subjects. Younger subjects were more aware of the risk factors of diabetes. Recognizing the presence of a family history as a significant risk factor for developing diabetes was infrequent among subjects, but more common among the educated and those with higher incomes. Those who reported a positive family history of diabetes were not much more aware of family history as a risk factor for DM than those with no positive family history for the disease.

**Table 2:** Knowledge of diabetes risk factors by demographic factors and family history of diabetes among non-diabetic respondents

	**Excessive sugar intake**		**Obesity**		**Physical inactivity**		**Presence of family history**	
	***%***	***p***	***%***	***p***	***%***	***p***	***%***	***p***

Gender								
Males	59.5	0.881	29.5	0.982	27.4	0.001	19.0	0.254
Females	60.1		29.4		16.0		15.3	

Age group								
20–30	64.4	0.007	35.6	0.001	22.5	0.021	23.2	0.001
31–50	58.8		25.7		23.0		11.8	
> 50	44.9		15.4		9.0		5.1	

Educational status								
Illiterate	50.0	0.0001	18.8	0.0001	12.5	0.0001	6.3	0.0001
< high school	50.8		24.9		16.9		9.0	
High school	69.5		31.7		19.8		18.6	
≥ Some college	72.6		47.4		40.0		42.1	

Monthly H. Hold Income								
OMR < 300	54.0	0.038	26.5	0.009	15.6	0.0001	13.7	0.015
300 – 1000	63.6		27.5		20.8		16.7	
> 1000	68.9		45.9		41.0		39.5	

Family History of Diabetes								
Positive History	63.4	0.067	30.0	.758	21.8	0.528	19.5	0.076
No/Not sure	55.8		28.8		19.6		13.8	

Total/combined	59.9%		29.5%		20.8%		16.9%	

p-values for comparisons of knowledge between the groups for each risk factor

Awareness of diabetes as a serious condition with resultant intransigent complications was suboptimal. Only 55.1% of the sample knew that diabetes is a condition that, if uncontrolled, can produce lifelong complications affecting different organs of the body. Visual problems were identified as complications by 24.3% of the subjects, followed by heart disease (20.4%), kidney disease (17.9%), stroke (9.4%) and delayed wound healing and complicated diabetic foot (6.0%).

Knowledge among non-diabetic respondents of common preventive measures, relative to socio-demographic factors, is shown in Table 3.

**Table 3:** knowledge of common preventive measures by demographic factors and family history of diabetes among non-diabetic respondents

	**Taking Care of the Diet**		**Increasing Physical Activity**		**Avoiding Obesity & overweight**	
	***%***	***p***	***%***	***p***	***%***	***p***

Gender						
Males	61.2	0.432	49.8	0.002	19.0	0.581
Females	64.4		36.8		17.2	

Age group						
20–30	67.4	0.0001	53.4	0.0001	23.2	0.001
31–50	67.4		36.4		13.9	
> 50	35.9		14.1		7.7	

Educational status						
Illiterate	38.4	0.0001	11.6	0.0001	8.9	0.004
< high school	61.4		33.9		15.3	
High school	74.3		56.3		24.6	
≥ Some college	75.8		70.5		22.1	

Monthly H. Hold Income						
OMR < 300	57.3	0.034	30.3	0.0001	14.2	0.087
300 – 1000	66.9		47.2		18.2	
> 1000	72.1		62.3		26.2	

Family History of Diabetes						
Positive History	66.8	0.117	46.2	0.042	19.5	0.306
No/Not sure	59.6		37.7		16.2	

Total/combined	63.1%		42.3%		17.9%	

p-values for comparisons of knowledge between the groups for each of the preventive measures

It can be seen that 63.1% reported that diabetes can be prevented by modifying dietary habits, and 42.3% felt that diabetes can be prevented by increasing physical activity; however, only 17.9% felt that avoiding obesity and reducing weight play important roles in the prevention of diabetes. Approximately 78.9% of the study sample perceived diabetes as a preventable condition. Awareness of increasing physical activity as a preventative measure was greater among male subjects (p < 0.005). Additionally, younger subjects and those with a higher level of education and higher monthly incomes were more aware of common preventative measures for diabetes.

Associated variables pertaining to knowledge about the definition of diabetes; its symptoms, risk factors, complications; and preventative measures are shown in Table 4.

**Table 4:** Predictors of knowledge of diabetes definition, symptoms, risk factors, complications and preventive measures based on fitting Stepwise multiple logistic regression models in the study groups

	**Definition**	**Symptoms**	**Risk factors**	**Complications**	**Prevention**
Demographic factor	OR (95% CI) p	OR (95% CI) p	OR (95% CI) p	OR (95% CI) p	OR (95% CI) p

**Education;**					
Less than high-school	1.00				
High-school & above	4.69 (3.22 – 6.82) 0.0001	3.27 (2.22 – 4.82) 0.0001	3.66 (2.37 – 5.65) 0.0001	1.93 (1.36 – 2.74) 0.0001	3.44 (2.19 – 5.41) 0.0001

**Family history of DM;**					
No/Don't know	1.00				
Yes	1.49 (1.03 – 2.16) 0.034	1.98 (1.36 – 2.88) 0.0001	1.51 (1.01 – 2.26) 0.044	2.10 (1.48 – 2.97) 0.0001	1.58 (1.05 – 2.37) 0.029

**Income**					
< 300 OMR p.m.	1.00				
≥ 300 OMR p.m.	1.48 (1.01 – 2.18) 0.047	----------	---------	---------	1.61 (1.07 – 2.44) 0.024

OR: Odds ratio; CI: Confidence interval; DM: Diabetes mellitus.

Age, sex, education, income and family history were entered into a multivariate logistic regression model for each potential predictor of knowledge about DM. However, age and sex were eliminated by the step-wise regression procedure. Analysis revealed that those with a high school education or higher were 4.69 times more likely to know the definition of diabetes than those with a lower level of education (95% CI 3.22 – 6.82; p < 0.001). Higher education level also was significantly associated with greater knowledge regarding diabetes symptoms (OR 3.27; 95% CI 2.22–4.82, p < 0.001)), risk factors (OR 3.66; 95% CI 2.37–5.65; p < 0.001), complications (OR 1.93; 95% CI 1.36–2.74; p < 0.001) and prevention (OR 3.44; 95% CI 2.19–5.41; p < 0.001). Positive family history of diabetes also predicted each of these response variables. However, subject income level only was significantly associated with knowledge regarding diabetes definition and prevention.

## Discussion

Oman has been successful in reducing the incidence of communicable diseases and increasing the standard of life among its people. Rapid cultural changes and social advances since 1970 have led to the manifestation of a wide range of non-communicable diseases. High prevalence rates for diabetes (11.6%), obesity (20.5%) and metabolic syndrome (21.0%) exist in the Omani population [20]. It is recognized that health promotion, based upon societal knowledge and perceptions regarding chronic diseases like diabetes, is an essential component of any strategy aimed at disease control and prevention.

In order to lay the groundwork for health education for emerging health problems, in this study, we surveyed a semi-urban population of Oman regarding its level of knowledge and overall perceptions of diabetes. The data are somewhat discouraging. More than half of the subjects (53.5%) were unable to provide even a rudimentary definition of diabetes, though they might be aware of its existence or have some idea about its symptoms or risk factors. Also, the study showed that knowledge regarding classic symptoms of diabetes was limited, and that two thirds of the subjects were unable to recognize obesity as a risk factor. Our results are not unlike those that have been reported elsewhere. Based upon the results of a survey they conducted in a metropolitan city in India, Mohan *et al*. reported that about one third of the general public was unaware of the term 'diabetes' [21]. The failure to define diabetes and to recognize its symptoms may reflect the general public's significant lack of knowledge about diabetes. This is likely to have negative repercussions, in terms of trying to control and prevent diabetes.

It is widely acknowledged that excessive sugar intake is a risk factor for incident diabetes mellitus [22]. Recent affluence in Oman has been marked by notable changes in dietary habits, which have included an increased likelihood of excessive sugar intake. Approximately 60% of the population surveyed perceived high consumption of dietary sugar as an important risk factor for developing diabetes. However, psychosocial studies consistently have shown that there is discrepancy between one's attitudes and behaviors [23]. Therefore, it remains to be seen whether knowledge regarding the adverse effects of excessive sugar intake will translate into personal decisions to curtail excessive sugar intake among Omanis.

The present study indicates that one's level of education has a direct influence on one's level of knowledge regarding the definition, symptoms, risk factors and complications of, and preventative measures against diabetes. This suggests that knowledge about diabetes is conducive to health education. This finding is congruent with other studies [24,25] albeit with a few exceptions [26]. In addition to education, a family history of diabetes also appears to influence one's level of knowledge and perceptions of diabetes. Individuals with a positive family history of a disease may develop a personal sense of vulnerability which, in turn, may increase their awareness, as was revealed in the present study [27]. Risk perception is an essential concept in a number of theoretical models addressing health-protective behaviors. Perceived risk is considered to be the primary motive to change within the *Health Belief Model*, which assumes that, the higher the perceived threat, the more likely an individual will modify his or her behavior to circumvent that threat [28,29]. A positive family history of a disease, and one's gender, age and perceptions of disease seriousness may affect one's level of perceived risk [30]. In support of this view, Harwell *et al *found that family history is the factor most significantly associated with the perceived risk of developing diabetes [31]. However, Pierce *et al*, in their randomized controlled trial, found that the family members of individuals with type 2 diabetes underestimate their own risk of developing the disease [32]. Factors influencing perceptions of family history may vary between individuals and between diseases. In the available literature and present findings, it has emerged that perceived risk may be important to motivate preventative health behaviors and control of disease [33].

One implication of the present findings is that, despite limited knowledge of diabetes, education that could be gleaned could play a critical role in coming to grips with the emerging epidemic of diabetes. More research is needed to identify how to increase public knowledge and perceptions of this disease.

## Limitations of the study

Some limitations of this study should be highlighted. First, the findings of this study cannot be generalized to all of Oman, as the data were derived from semi-urban satellite towns adjacent to the capital of Muscat. However, the present findings lay the groundwork for further similar studies in other parts of the country. Second, to accommodate individuals who might be illiterate, the items were read to the subjects, rather than allowing them to self-administer the questionnaire. It is possible that this approach might have resulted in subject reluctance to reveal sensitive feelings that may have been more fully elicited in a self-administered questionnaire. Having said this, the questionnaire was specifically devised not to pry into people's private lives; consequently, there is no explicit reason to suspect that subjects would be reluctant to respond honestly. Moreover, one confounding variable on this approach is the likelihood of lack of consistency in reading out the questions to the subjects bearing in mind that there were several different researchers dispending the questionnaire. Although this confounding factor remains, during the preparation of the study there was an acceptable inter-coding agreement. There also tends to be culturally-specific responses to questionnaires [34], a potential bias that was not explored in the present study, and which might have played a role, given that the study relied almost exclusively on self-reported, subjective data. These limitations and countless others that were not apparent, but are yet common in psychosocial studies, suggest that extrapolating the present findings to other populations should be viewed with caution.

## Conclusion

The central objectives in this research were (1) to assess general population knowledge and perceptions of diabetes, and (2) to tease out whatever relationships that exist between the various components of knowledge and socio-demographic background in a semi-urban community in Oman. This study has demonstrated that significant numbers of Omanis lack the knowledge and perceptions required to prevent and cope with the increasing prevalence of diabetes in Oman. On the bright side, the study strongly implicates level of education as the most significant predictor of desirable knowledge and perceptions of diabetes risk factors, complications and prevention. This raises optimism that health education could be a powerful tool as we strive to develop strategies to fight debilitating and rapidly growing public health problems in Oman, problems that often are amenable to life style changes and, by implication, education.

## Competing interests

The authors declare that they have no competing interests.

## Authors' contributions

MAAS conceived and designed the study, analysed the data and drafted and finalized the manuscript. SAS participated in the design of the study, data analysis and the interpretation of results. SGAR participated in data analysis. YAF participated in the design of the study. MAK participated in statistical analysis and in manuscript revision. SSG and MA participated by scrutinizing the statistics in the tables and the results, and by providing assistance to address reviewers' comments. SAA participated by rewriting draft versions and critically revising the manuscript. All authors read and approved the final manuscript.

## Pre-publication history

The pre-publication history for this paper can be accessed here:


